# Mammalian orthoreovirus capsid protein σ3 antagonizes RLR-mediated antiviral responses by degrading MAVS

**DOI:** 10.1128/msphere.00236-24

**Published:** 2024-05-17

**Authors:** Dianyu Li, Rongqian Mo, Xiaoyi Li, Rongrong Cheng, Jingying Xie, Hongshan Li, Yanmei Yang, Shasha Li, Huixia Li, Zhenfang Yan, Suocheng Wei, Adi Idris, Xiangrong Li, Ruofei Feng

**Affiliations:** 1 Key Laboratory of Biotechnology and Bioengineering of State Ethnic Affairs Commission, Biomedical Research Center, Northwest Minzu University, Lanzhou, China; 2 Gansu Tech Innovation Center of Animal Cell, Biomedical Research Center, Northwest Minzu University, Lanzhou, China; 3 College of Life science and Engineering, Northwest Minzu University, Lanzhou, China; 4 Engineering Research Center of Key Technology and Industrialization of Cell-based Vaccine, Ministry of Education, Biomedical Research Center, Northwest Minzu University, Lanzhou, China; 5 Centre for Immunology and Infection Control, School of Biomedical Sciences, Queensland University of Technology, Kelvin Grove, Queensland, Australia; Johns Hopkins University Bloomberg School of Public Health, Baltimore, Maryland, USA

**Keywords:** mammalian orthoreovirus, σ3 protein, RIG-I-like receptor signaling, mitochondrial antiviral signaling protein

## Abstract

Mammalian orthoreovirus (MRV) outer capsid protein σ3 is a multifunctional protein containing a double-stranded RNA-binding domain, which facilitates viral entry and assembly. We reasoned that σ3 has an innate immune evasion function. Here, we show that σ3 protein localizes in the mitochondria and interacts with mitochondrial antiviral signaling protein (MAVS) to activate the intrinsic mitochondria-mediated apoptotic pathway. Consequently, σ3 protein promotes the degradation of MAVS through the intrinsic caspase-9/caspase-3 apoptotic pathway. Moreover, σ3 protein can also inhibit the expression of the components of the RNA-sensing retinoic acid-inducible gene (RIG)-like receptor (RLR) signaling pathway to block antiviral type I interferon responses. Mechanistically, σ3 inhibits RIG-I and melanoma differentiation-associated gene 5 expression is independent of its inhibitory effect on MAVS. Overall, we demonstrate that the MRV σ3 protein plays a vital role in negatively regulating the RLR signaling pathway to inhibit antiviral responses. This enables MRV to evade host defenses to facilitate its own replication providing a target for the development of effective antiviral drugs against MRV.

**IMPORTANCE:**

Mammalian orthoreovirus (MRV) is an important zoonotic pathogen, but the regulatory role of its viral proteins in retinoic acid-inducible gene-like receptor (RLR)-mediated antiviral responses is still poorly understood. Herein, we show that MRV σ3 protein co-localizes with mitochondrial antiviral signaling protein (MAVS) in the mitochondria and promotes the mitochondria-mediated intrinsic apoptotic pathway to cleave and consequently degrade MAVS. Furthermore, tryptophan at position 133 of σ3 protein plays a key role in the degradation of MAVS. Importantly, we show that MRV outer capsid protein σ3 is a key factor in antagonizing RLR-mediated antiviral responses, providing evidence to better unravel the infection and transmission mechanisms of MRV.

## INTRODUCTION

Mammalian orthoreovirus (MRV) belongs to the *Orthoreovirus* genus of the *Spinareoviridae* family, which is widespread in nature and can infect almost all mammals, including humans (1). MRV belongs to a class of replication-competent oncolytic viruses, a characteristic attributed to its transcriptional efficiency (2, 3). MRV is a non-enveloped double-stranded RNA (dsRNA) virus of approximately 70 nm in diameter. MRV genome encodes 12 proteins, including nine structural (λ1, λ2, λ3, μ1, μ1C, μ2, σ1, σ2, and σ3) and three non-structural proteins (4). MRV infection requires the synergistic action of viral and host factors to facilitate its entry into cells. Upon entry, structural protein σ1 interacts with cell surface carbohydrates and/or protein receptors to facilitate its internalization. Specifically, α-linked sialic acid, junctional adhesion molecule A, Nogo-66 receptor 1, and paired immunoglobulin-like receptor B have been described as host entry receptors for MRV (5–7). MRV S1 and S4 genes that encode σ1 protein and σ3 protein, respectively, both form a heterohexamer that surrounds the core of the virion (8, 9) and constitutes the core outer layer protein of MRV capsid structure. Importantly, σ3 possesses an RNA-binding domain (RBD) and a zinc-finger domain, and has been shown to prevent the activation of dsRNA-activated protein kinase R (PKR) response (10, 11). PKR is a critical player in the host antiviral response and exerts its antiviral function by blocking host translation machinery and induces cellular apoptosis. In short, σ3 protein is a virulence factor that suppresses PKR activation and subsequent stress granule formation. σ3 has also been shown to block nuclear factor kappa-B (NF-κB)-dependent host antiviral machinery to dampen inflammatory factors and type I interferons (IFNs) (12, 13), albeit a poorly elucidated mechanism.

The innate immune system is the host’s first line of defense against pathogens. Host pattern recognition receptors (PRRs), including Toll-like receptors and the RNA-sensing retinoic acid-inducible gene (RIG)-like receptors (RLRs), defend against viral infections by recognizing pathogen-associated molecular patterns, such as viral nucleic acids or protein structures. Upon detection, antiviral responses are promptly activated through the stimulation of type I IFNs and other inflammatory factors (14, 15). RLR-sensing members, RIG-I and melanoma differentiation-associated gene 5 (MDA5), both recognize viral dsRNA and recruit mitochondrial antiviral signaling protein (MAVS) to successively activate the adaptor protein, TANK-binding kinase 1 (TBK1), and the nuclear translocation of IFN regulatory factor 3 (IRF3) to induce type I IFN (I-IFN) antiviral responses (16, 17). As a mitochondrial membrane protein, MAVS not only modulates I-IFN responses, but also participates in host cellular autophagy and apoptotic machineries (18, 19). Importantly, MRV is known to be sensed by the RIG-I/MDA5-sensing machinery (20). Given that MRV can antagonize the dsRNA-activated PKR response via σ3 protein (10, 11), we reasoned that MRV σ3 can also negatively regulate the RNA-sensing RIG-I pathway as an immune evasive strategy.

In this study, we elucidated the mechanism by which MRV σ3 protein negatively regulates the RLR signaling pathway to promote the replication of MRV and other RNA viruses *in vitro*. Moreover, we identified the RLR signaling pathway components targeted by σ3 protein for degradation. Our study provides a comprehensive account and mechanism by which MRV infection evades the host RLR-sensing machinery to aid its own survival.

## RESULTS

### MRV σ3 protein inhibits type I IFN responses triggered in cells infected with RNA viruses

**Fig 1:**
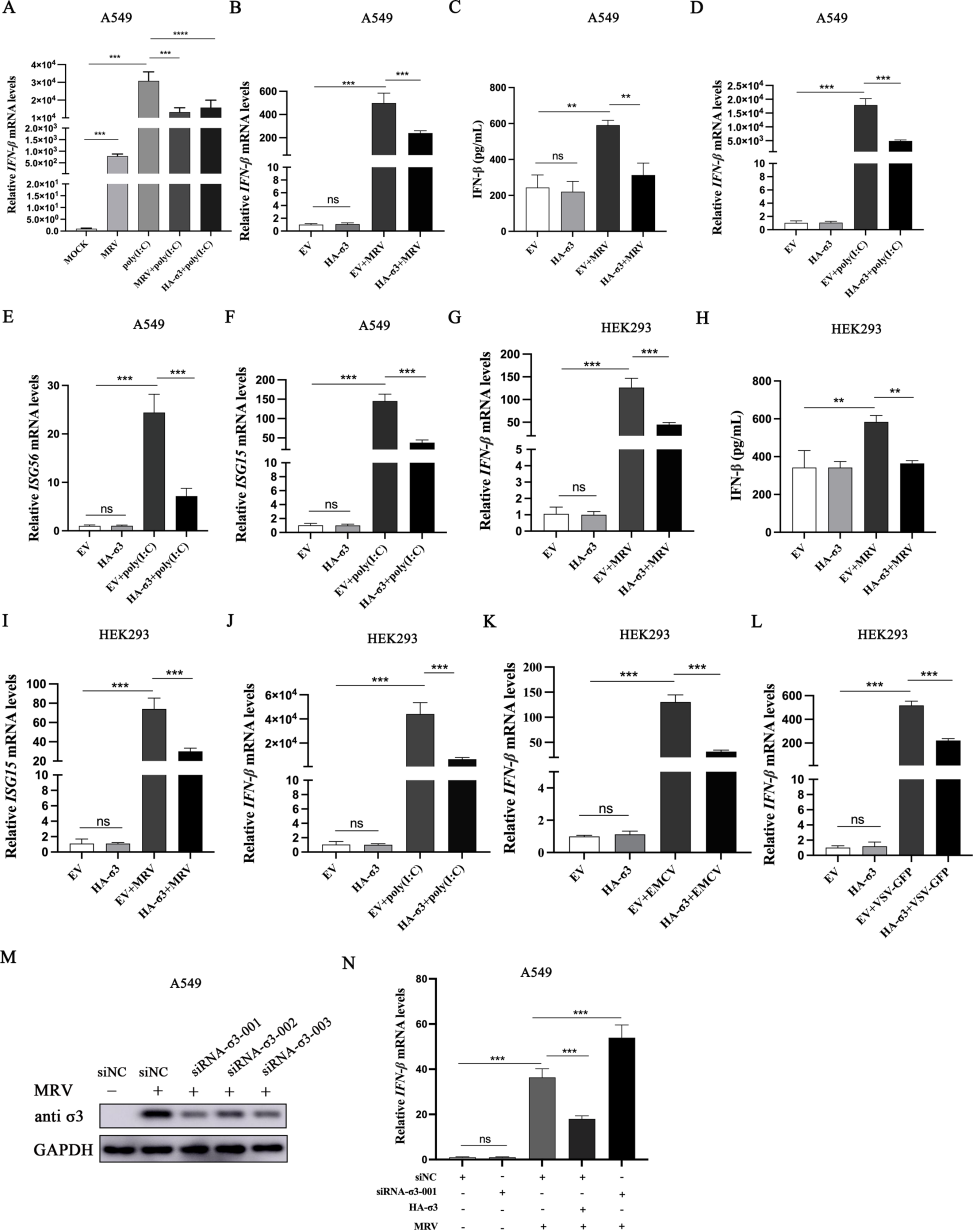
MRV σ3 protein inhibits the RNA viruses or poly(I:C)-induced activation of IFN-β and ISGs. (**A**) A549 cells were infected with MRV (multiplicity of infection [MOI] = 1) or transfected with 1 µg of HA-σ3 plasmid for 12 h, and then transfected with 2 µg of poly(I:C) for 12 h. Quantitative real-time PCR (qPCR) was utilized detect the mRNA expression level of *IFN-*β. (**B**) A549 cells were transfected with 1 µg of EV or HA-σ3 plasmid for 24 h followed by MRV infection (MOI = 0.01) for 12 h. qPCR was used to detect the mRNA expression level of *IFN-*β. (**C**) A549 cells were transfected with 1.0 µg of EV or HA-σ3 plasmid for 24 h followed by MRV infection (MOI = 0.01) for 24 h. Human IFN-β enzyme-linked immunosorbent assay (ELISA) Kit was used to detect the production of IFN-β in cell supernatants. (**D–F**) A549 cells were transfected with 1 µg of EV or HA-σ3 plasmid for 24 h followed by poly(I:C) transfection for 12 h. qPCR was used to detect the mRNA expression level of *IFN-*β, *ISG56*, and *ISG15*. (**G**) HEK293 cells were transfected with 1 µg of EV or HA-σ3 plasmid for 24 h followed by MRV infection (MOI = 0.01) for 12 h. qPCR was used to detect the mRNA expression level of *IFN-*β. (**H**) HEK293 cells were transfected with 1 µg of EV or HA-σ3 plasmid for 24 h followed by MRV infection (MOI = 0.01) for 24 h. Human IFN-β ELISA Kit was used to detect the production of IFN-β in cell supernatants. (**I**) HEK293 cells were transfected with 1 µg of EV or HA-σ3 plasmid for 24 h followed by MRV infection (MOI = 0.01) for 12 h. qPCR was used to detect the mRNA expression level of *ISG15*. (**J**) HEK293 cells were transfected with 1 µg of EV or HA-σ3 plasmid for 24 h followed by poly(I:C) transfection for 12 h. qPCR was used to detect the mRNA expression level of *IFN-*β. (**K**) HEK293 cells were transfected with 1 µg of EV or HA-σ3 plasmid for 24 h followed by EMCV infection (MOI = 0.001) for 12 h. qPCR was used to detect the mRNA expression level of *IFN-*β. (**L**) HEK293 cells were transfected with 1 µg of EV or HA-σ3 plasmid for 24 h followed by VSV-GFP infection (MOI = 0.001) for 12 h. qPCR was used to detect the mRNA expression level of *IFN-*β. (**M**) A549 cells were transiently transfected with 150 nm non coding small interfering RNA (siNC), small interfering RNA (siRNA) -σ3-001, siRNA-σ3-002, or siRNA-σ3-003 for 12 h followed by MRV infection (MOI = 0.01) for 24 h. Western blotting was used to detect the expression of endogenous σ3 protein. Glyceraldehyde-3-phosphate dehydrogenase (GAPDH) was used as a loading control. (**N**) A549 cells were transiently transfected with 150 nm siNC or siRNA-σ3-001 for 12 h followed by 1 µg of EV or HA-σ3 transfection for 12 h. Then, these cells were infected with MRV (MOI = 0.01) for 24 h. qPCR was used to detect the mRNA expression level of *IFN-*β. The data were represented as the mean ± SD of three independent experiments. The measurements were performed in technical duplicate. Statistical significance was denoted as **P < 0.01 and ***P < 0.001.

Given that MRV σ3 protein is imbued with an RBD, we hypothesize that this protein can antagonize a host RNA-sensing machinery and subsequently dampen I-IFN responses. Cells stimulated with a synthetic dsRNA, polyinosinic-polycytidylic acid [poly(I:C)], a known cytosolic receptor MDA5 and RIG-I trigger (21), rapidly induced an IFN-β response, which was blocked in the presence of an MRV infection (Fig. 1A), suggesting that MRV is antagonizing this response. Moreover, under the same conditions, the heterologous expression of σ3 protein also blocked poly(I:C)-activated IFN-β production (Fig. 1A). As we hypothesized, we showed that exogenous overexpression of MRV σ3 significantly inhibited IFN-β responses and IFN-stimulated genes (ISG) expression triggered by either MRV infection or poly(I:C) in two independent cell lines, A549 cells (Fig. 1B through F) and HEK293 cells (Fig. 1G through J). Importantly, this observed antagonistic response was also observed in cells infected by other RNA viruses known to activate I-IFN responses (22, 23) (i.e., encephalomyocarditis virus [EMCV] and vesicular stomatitis virus [VSV] tagged with green fluorescent protein [GFP]) (Fig. 1K and L), suggesting that MRV σ3 is blocking a host RNA-sensing pathway, possibly the RIG-I/MDA5 pathway. To confirm this observation with endogenous MRV σ3 protein, we genetically silenced σ3 expression in MRV-infected cells using RNA interference (RNAi) (Fig. 1M). Indeed, RNAi-mediated silencing of endogenous σ3 restored MRV-mediated blockade of IFN-β responses (Fig. 1N), further confirming the antagonistic function of σ3 on I-IFN responses.

### MRV σ3 protein enhances MRV, EMCV, and VSV replication

**Fig 2:**
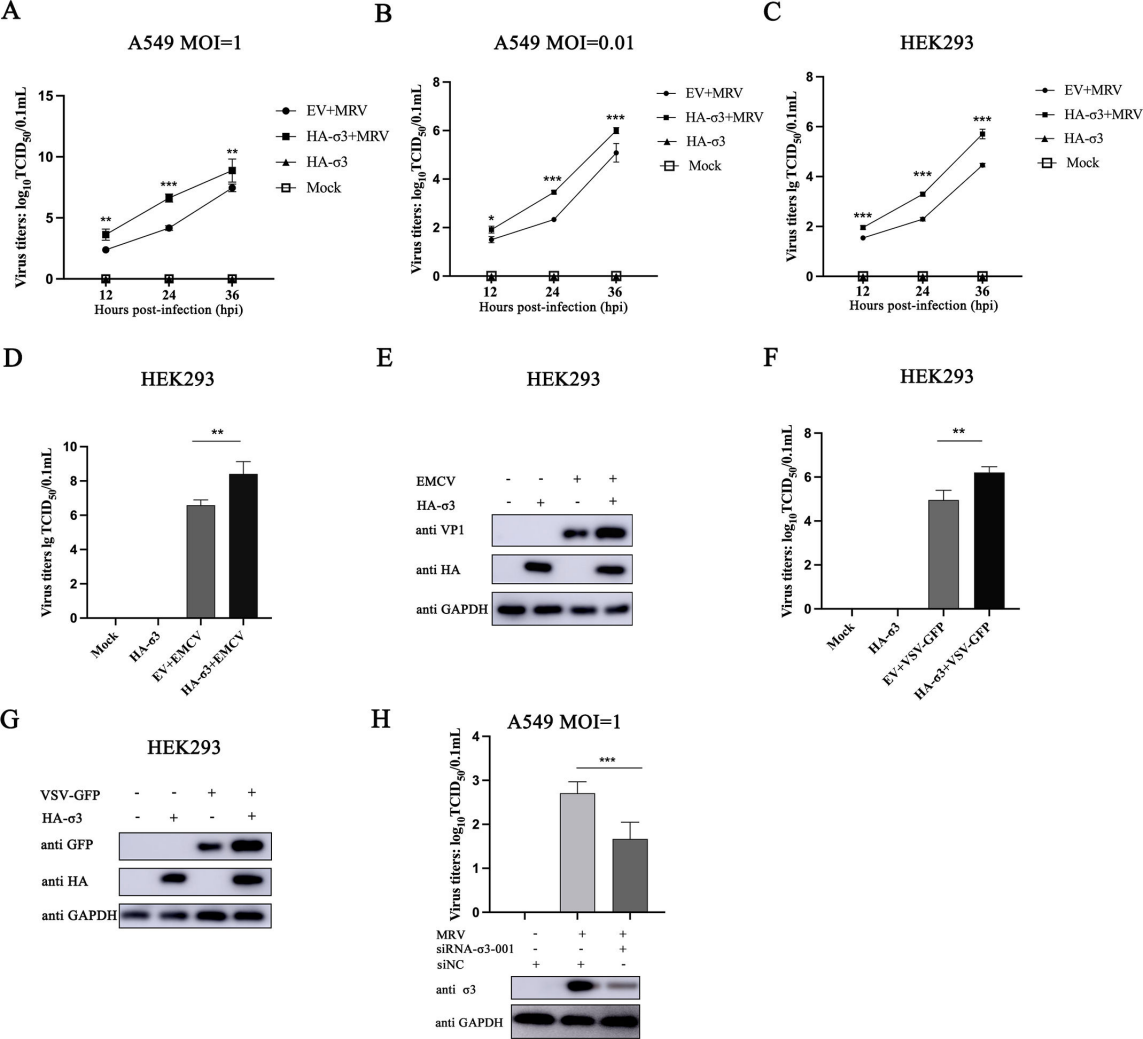
The σ3 protein promotes the replication of MRV, EMCV, and VSV *in vitro*. (**A**) A549 cells were transfected with 1 µg of EV or HA-σ3 plasmid for 12 h followed by MRV infection (MOI = 1) for 12, 24 or 36 h. A 50% tissue culture infective dose (TCID_50_) (Reed-Muench method) was used to detect MRV titers. (B and C) A549 cells or HEK293 cells were transfected with 1 µg of EV or HA-σ3 plasmid for 24 h followed by MRV infection (MOI = 0.01) for 12, 24 or 36 h. TCID_50_ assay was used to detect MRV titers. (D) HEK293 cells were transfected with 1 µg of EV or HA-σ3 plasmid for 24 h followed by EMCV infection (MOI = 0.001) for 24 h. TCID_50_ assay was used to detect EMCV titers. (E) HEK293 cells were transfected with 1 µg of EV or HA-σ3 plasmid for 24 h followed by EMCV infection (MOI = 0.001) for 24 h. Western blotting was used to detect the expression of HA-tagged σ3 and viral VP1 protein, respectively. GAPDH was used as a loading control. (F) HEK293 cells were transfected with 1 µg of EV or HA-σ3 plasmid for 24 h followed by VSV-GFP infection (MOI = 0.001) for 24 h. TCID_50_ assay was used to detect VSV titers. (G) HEK293 cells were transfected with 1 µg of EV or HA-σ3 plasmid for 24 h followed by VSV-GFP infection (MOI = 0.001) for 24 h. Western blotting was used to detect the expression of HA-tagged σ3 and GFP, respectively. GAPDH was used as a loading control. (H) A549 cells were transiently transfected with 150 nm siNC or siRNA-σ3-001 for 12 h followed by MRV infection (MOI = 1) for 12 h. TCID_50_ assay was used to detect MRV titers. Western blotting was used to detect the expression of viral σ3 protein. GAPDH was used as a loading control. The data were represented as the mean ± SD of three independent experiments. The measurements were performed in technical duplicate. Statistical significance was denoted as *P < 0.05, **P < 0.01, and ***P < 0.001.

Given that MRV σ3 has IFN-antagonistic effects, we expect that this would create a conducive environment for viruses to replication in cells. Indeed, exogenous expression of MRV σ3 protein significantly enhanced MRV replication in A549 cells (Fig. 2A and B) and HEK293 cells (Fig. 2C), irrespective of the virus infective load (multiplicity of infection (MOI)). To validate whether MRV σ3 protein has any effect on the replication of other RNA viruses, HEK293 cells overexpressing σ3 were infected with either EMCV or VSV-GFP. Overexpression of σ3 significantly increased both EMCV and VSV-GFP infectious titers *in vitro*, correlating with an increase in VP1 and GFP protein expression, respectively (Fig. 2D through G). Consistent with this, MRV titer was significantly decreased when σ3 expression was knocked down (Fig. 2H), indicating that the σ3 protein has a positive regulatory effect on RNA virus replication *in vitro*.

### MRV σ3 protein inhibits the expression of RLR signaling cascade mediators

**Fig 3:**
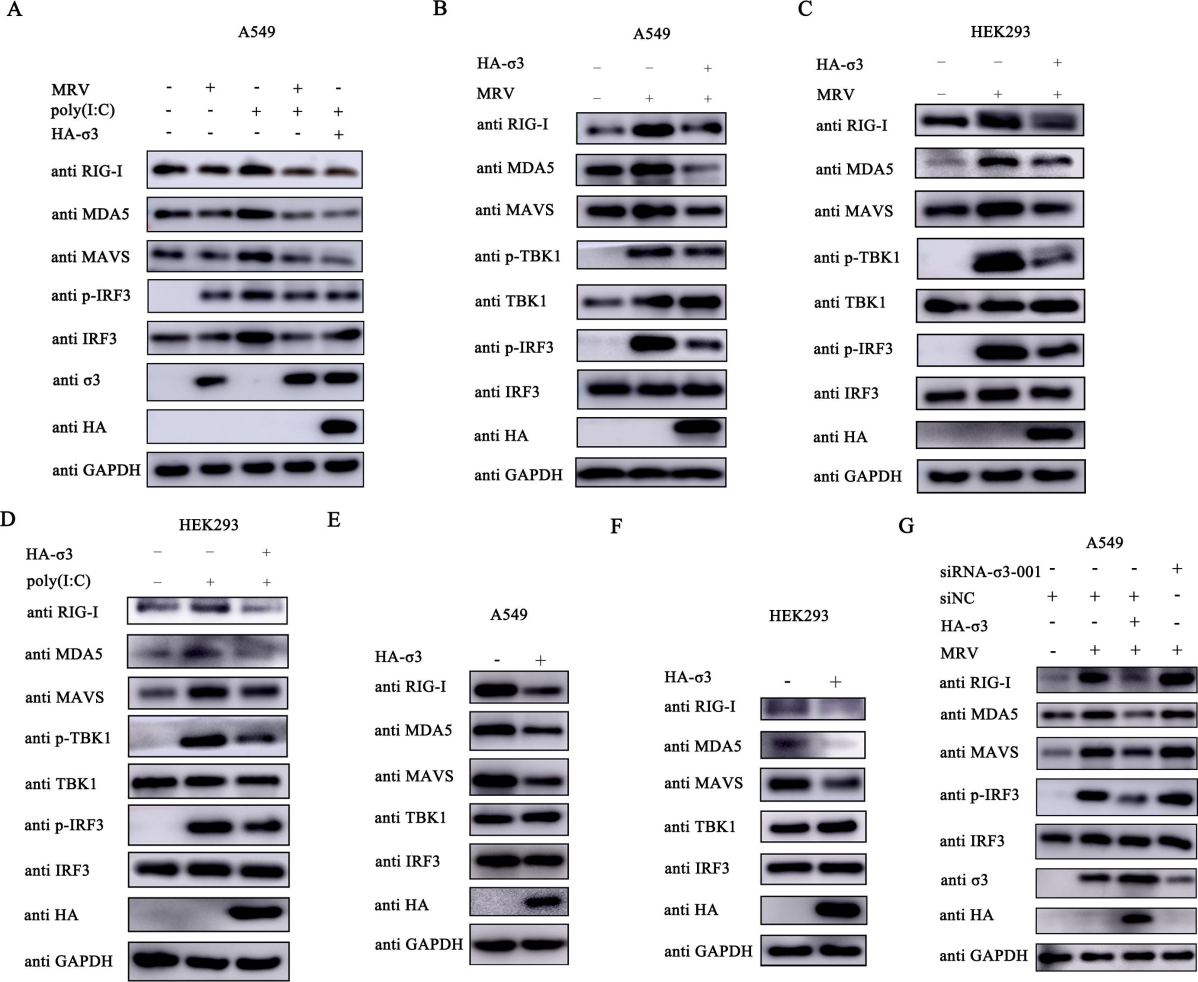
MRV σ3 protein inhibits the protein expression of RIG-I, MDA5, and MAVS. (**A**) A549 cells were infected with MRV (MOI = 1) or transfected with 1 µg of HA-σ3 plasmid for 12 h, and then transfected with 2 µg of poly(I:C) for 12 h. Western blotting was used to detect the protein expression of RIG-I, MDA5, MAVS, IRF3, p-IRF3, viral σ3, and HA-tagged-σ3, respectively. GAPDH was used as a loading control. (B and C) A549 cells or HEK293 cells were transfected with 1 μg of EV or HA-σ3 plasmid for 24 h followed by MRV infection (MOI = 0.01) for 24 h. Western blotting was used to detect the protein expression of RIG-I, MDA5, MAVS, TBK1, p-TBK1, IRF3, p-IRF3, and HA-tagged-σ3, respectively. GAPDH was used as a loading control. (D) HEK293 cells were transfected with 1 µg of EV or HA-σ3 plasmid for 24 h followed by poly(I:C) transfection for 12 h. Western blotting was used to detect the protein expression of RIG-I, MDA5, MAVS, TBK1, p-TBK1, IRF3, p-IRF3, and HA-tagged-σ3, respectively. GAPDH was used as a loading control. (E and F) A549 cells or HEK293 cells were transfected with 1 µg EV or HA-σ3 plasmid for 24 h. Western blotting was used to detect the protein expression of endogenous RIG-I, MDA5, MAVS, TBK1, IRF3, and HA-tagged-σ3, respectively. GAPDH was used as a loading control. (G) A549 cells were transiently transfected with 150 nm siNC or siRNA-σ3-001 for 12 h followed by 1 µg of EV or HA-σ3 transfection for 12 h. Then these cells were infected with MRV (MOI = 0.01) for 24 h. Western blotting was used to detect the expression of RIG-I, MDA5, MAVS, IRF3, p-IRF3, HA-tagged-σ3, and viral σ3 protein, respectively. GAPDH was used as a loading control.

MRV is known to be sensed by the RIG-I/MDA5 sensing machinery (20). Considering our findings so far, we surmise that MRV σ3 protein may be inhibiting I-IFN responses by antagonizing the functions of key adaptor molecules in the RLR signaling pathway. To interrogate this, cells infected with MRV or overexpressing σ3 were stimulated with poly(I:C) before immunoblotting for RLR signaling pathway proteins. Similar to the observations with exogenously expressed σ3, MRV infection with a high MOI antagonized poly(I:C)-induced expression of RIG-I, MDA5, MAVS, and p-IRF3 (Fig. 3A). We also found that exogenous expression of MRV σ3 inhibited the expression of RIG-I, MDA5, MAVS, p-TBK1, and p-IRF3 induced by MRV or poly(I:C) alone (Fig. 3B through D). Moreover, overexpression of MRV σ3 alone in the absence of RLR stimulation also dampened the expression of these signaling proteins (Fig. 3E and F). Importantly, siRNA-mediated knockdown of MRV σ3 restored the expression of RIG-I, MDA5, MAVS, and p-IRF3 (Fig. 3G), confirming that MRV σ3 protein interferes with RLR signaling components.

### MRV σ3 protein inhibits the transduction of RLR signaling pathway by targeting MAVS

**Fig 4:**
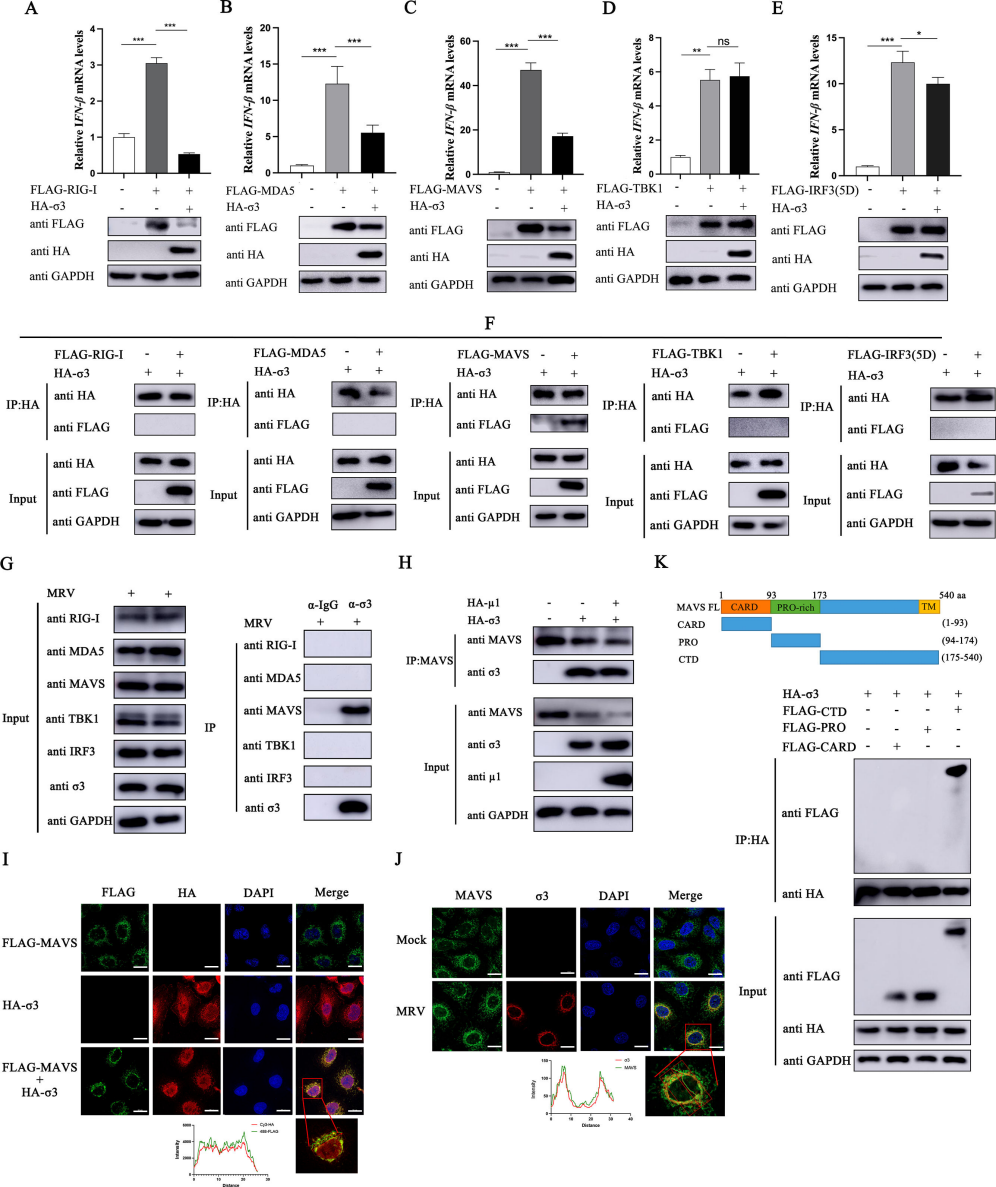
The σ3 protein interacts with MAVS and the C-terminal domain of MAVS is essential for the interaction. (**A-E**) HEK293 cells were co-transfected with 1.0 µg of EV or HA-σ3 plasmid and 1.0 µg of FLAG-RIG-I, FLAG-MDA5, FLAG-MAVS, FLAG-TBK1, or FLAG-IRF3(5D) for 24 h, respectively. Total RNA was extracted and qPCR (upper panel) was used to detect the mRNA expression level of *IFN-*β. Western blotting (lower panel) was used to detect the expression of HA-tagged σ3 protein and FLAG-tagged RIG-I, MDA5, MAVS, TBK1, or IRF3 proteins, respectively. GAPDH was used as a loading control. (**F**) HEK293 cells were co-transfected with 1.0 µg of HA-σ3 plasmid and 1.0 µg of pCMV-FLAG, FLAG-RIG-I, FLAG-MDA5, FLAG-MAVS, FLAG-TBK1, or FLAG-IRF3(5D) for 24 h, respectively. The cells were collected and lysed, protein G was added to adsorb the proteins in the cell lysate, and then immunoprecipitated with HA antibody. Western blotting was used to detect HA-tagged σ3 protein and FLAG-tagged RIG-I, MDA5, MAVS, TBK1, or IRF3 proteins in the whole cell lysates (Input) and immunoprecipitation (IP) complexes, respectively. (**G**) A549 cells were infected with MRV (MOI = 0.1) for 18 h, the cells were collected and lysed, protein G was added to adsorb the proteins in the cell lysate, and then immunoprecipitated with murine-derived IgG antibody or murine-derived reovirus σ3 antibody. Western blotting was used to detect the expression of σ3 protein and endogenous RIG-I, MDA5, MAVS, TBK1, and IRF3 proteins in the Input and IP complexes, respectively. (**H**) A549 cells were co-transfected with 1.0 µg of EV or 1.0 µg of HA-σ3 plasmid and 1.0 µg of HA-μ1 plasmid for 24 h, respectively. Then cells were collected and lysed, protein G was added to adsorb the proteins in the cell lysate, and then immunoprecipitated with MAVS antibody. Western blotting was used to detect the protein expression of MAVS, σ3 and μ1 in the Input and IP complexes, respectively. (**I**) A549 cells were transfected with either 1.0 µg of HA-σ3, FLAG-MAVS, or HA-σ3 + FLAG MAVS for 24 h before fixing cells and adding with corresponding antibodies for immunofluorescence imaging under a confocal microscope for HA (red), nucleus (blue), and FLAG (green). Scale bar = 20 µm. (**J**) A549 cells were infected with MRV (MOI = 0.01) for 24 h before fixing cells and adding with corresponding antibodies for immunofluorescence imaging under a confocal microscope for σ3 (red), nucleus (blue), and MAVS (green). Scale bar = 20 µm. (**K**) A549 cells were co-transfected with 1.0 µg of pCMV-FLAG, FLAG-CARD, FLAG-PRO, or FLAG-CTD plasmids and HA-σ3 plasmid for 24 h, respectively. Then, cells were collected and lysed, protein G was added to adsorb the proteins in the cell lysate, and then immunoprecipitated with HA antibody. Western blotting was used to detect the protein expression of HA-tagged σ3 protein and FLAG-tagged CARD, PRO, and CTD in the Input and IP complexes, respectively. The data were represented as the mean ± SD of three independent experiments. The measurements were performed in technical duplicate. Statistical significance was denoted as *P < 0.05, **P < 0.01, and ***P < 0.001.

To assess whether the observed dampening of the expression of RLR components is due to direct interaction with MRV σ3, we performed co-immunoprecipitation (Co-IP) experiments. Our analysis revealed that σ3 significantly inhibited RIG-I, MDA5, and MAVS-induced IFN-β activation and its protein expression (Fig. 4A through E). Interestingly, HA-tagged σ3 protein was found to only interact with FLAG-tagged MAVS and not the other RLR components (Fig. 4F). To substantiate this in an infection setting, we observed that σ3 interacted directly only with MAVS, not other RLR components during an MRV infection (Fig. 4G). Moreover, we found that HA-tagged σ3 can still be precipitated by magnetic beads coated with MAVS antibody in cells co-transfected with HA-tagged μ1 (Fig. 4H), suggesting that the σ3-MAVS interaction is specific. Indeed, by direct immunofluorescence microscopy, both exogenous σ3 and MAVS co-localizes (Fig. 4I). The heterologous expression of σ3 was mainly distributed in the nucleus, with some diffuse distribution in the cytosol. However, during an MRV infection, σ3 was mainly distributed in the perinuclear and cytosolic regions (Fig. 4J). This may be due to the interaction between the σ3 protein and μ1/μ1C protein to form a complex that blocks the migration of σ3 into the nucleus (24). Nonetheless, σ3 protein remains co-localized with MAVS. Next, we wanted to identify which region of MAVS is interacting with σ3. When we performed Co-IP of σ3 with various truncated versions of MAVS, only FLAG-tagged MAVS containing the C-terminal motif of MAVS was pulled down (Fig. 4K), suggesting that this motif containing the transmembrane domain is a critical region for this interaction. Taken together, these results demonstrate that σ3 protein interacts with MAVS and that the C-terminal domain of MAVS is essential for this interaction.

### Inhibition of RIG-I and MDA5 by MRV σ3 is independent of MAVS

**Fig 5:**
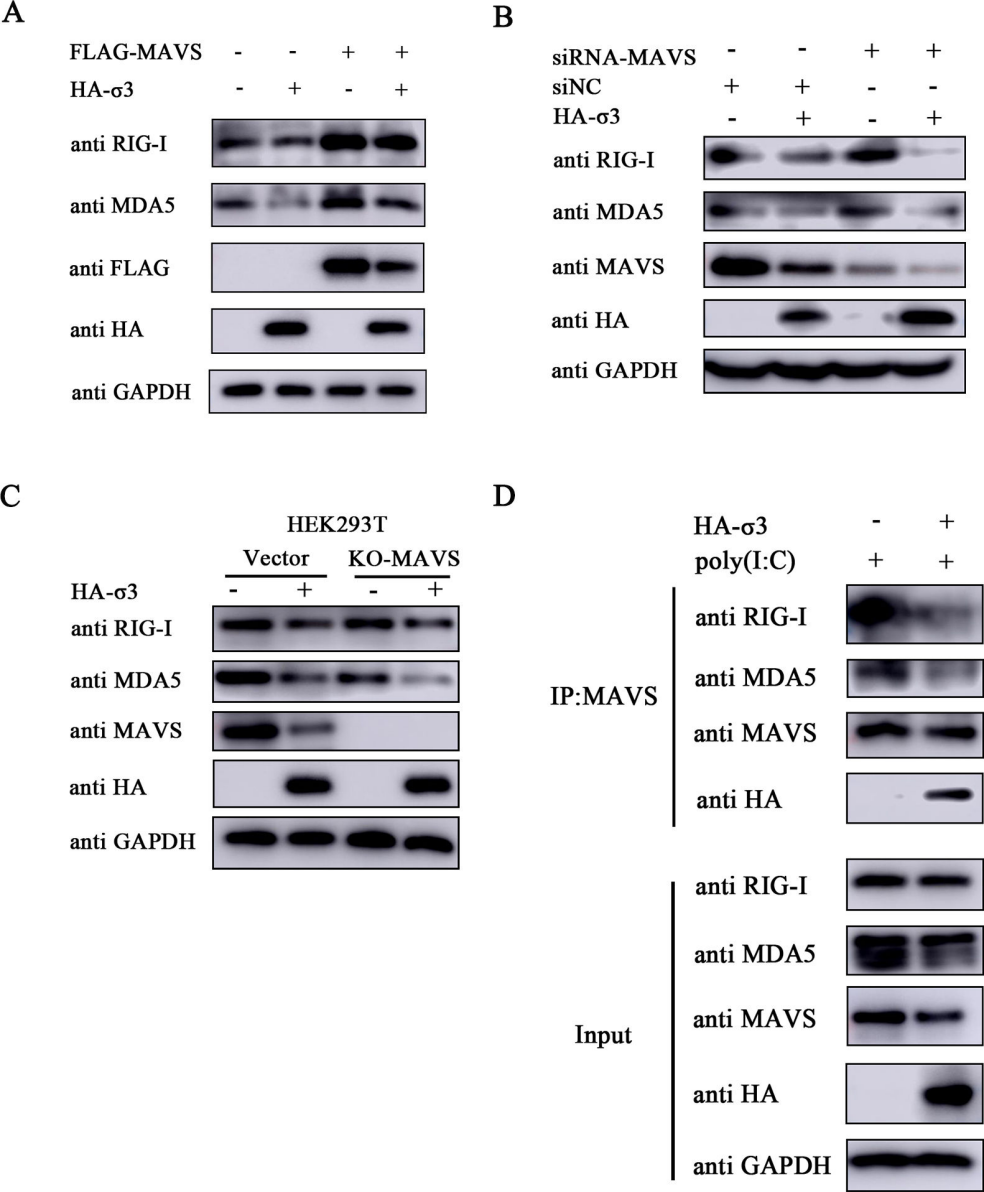
σ3 protein inhibits RIG-I and MDA5 expression is independent of its inhibitory effect on MAVS. (**A**) A549 cells were transfected with 1.0 µg of EV or HA-σ3 plasmid and 1.0 µg of pCMV-FLAG or FLAG-MAVS for 24 h, respectively. Western blotting was used to detect the protein expression of RIG-I, MDA5, FLAG-tagged MAVS, and HA-tagged σ3, respectively. GAPDH was used as a loading control. (**B**) A549 cells were transfected with 1.0 µg of EV or HA-σ3 plasmid for 24 h followed by 150 nM siRNA-MAVS or siNC transfection for 24 h. Western blotting was used to detect the protein expression of RIG-I, MDA5, MAVS, and HA-tagged σ3, respectively. GAPDH was used as a loading control. (**C**) HEK293T-Vector cells and HEK293T-KO-MAVS cells were transfected with either 1.0 µg of EV or HA-σ3 plasmid for 24 h, respectively. Western blotting was used to detect the protein expression of RIG-I, MDA5, MAVS, and HA-tagged σ3, respectively. GAPDH was used as a loading control. (**D**) A549 cells were transfected with either 1.0 µg of EV or HA-σ3 plasmid for 12 h, followed by transfection with 2.0 µg of poly(I:C) for another 12 h. Then cells were collected and lysed, protein G was added to adsorb the proteins in the cell lysate, and then immunoprecipitated with MAVS antibody. Western blotting was used to detect the protein expression of RIG-I, MDA5, MAVS, and HA-tagged σ3 in the Input and IP complexes, respectively.

Given that σ3 inhibited the expression of both RIG-I and MDA5 devoid of direct interaction (Fig. 4F), and that MAVS is known to have a mutual regulatory effect with RIG-I and MDA5 (25), we speculate that the inhibition of MAVS expression by σ3 blocks the transduction of the RLR signaling pathway, thereby indirectly leading to the downregulation of RIG and MDA5. Indeed, both RIG-I and MDA5 were upregulated in cells overexpressing MAVS alone (Fig. 5A), confirming the positive regulatory role of MAVS on these key RLR components. However, this was reversed when both MAVS and σ3 were overexpressed together suggesting that σ3 is acting in an antagonistic fashion. Overexpression of σ3 alone inhibited the expression of RIG-I, MDA5, and MAVS, whereas the inhibitory effect of σ3 on RIG-I and MDA5 remained when MAVS was genetically knocked down (Fig. 5B). Notably, exogenous expression of σ3 protein further suppressed RIG-I and MDA5 expression in cells which MAVS have been genetically silenced. These findings were corroborated in a cell line where MAVS have been stably knocked out (Fig. 5C). Biochemically, this suggests that the inhibition of RIG-I and MDA5 by MRV σ3 is occurring independently of MAVS. Nevertheless, it has been reported that RIG-I and MDA5 could recruit MAVS after the activation of the RLR pathway (26). We then wanted to see whether σ3 can influence this interplay between RIG-I or MDA5 and MAVS. Indeed, in the presence of poly I:C stimulation we observed that σ3 inhibited the interaction of both RIG-I and MDA5 with MAVS (Fig. 5D). Overall, these results suggest that σ3-mediated inhibition of RIG-I and MDA5 expression is occurring independently of its inhibitory effect on MAVS.

### MRV σ3 protein degrades MAVS through the intrinsic apoptotic pathway

**Fig 6:**
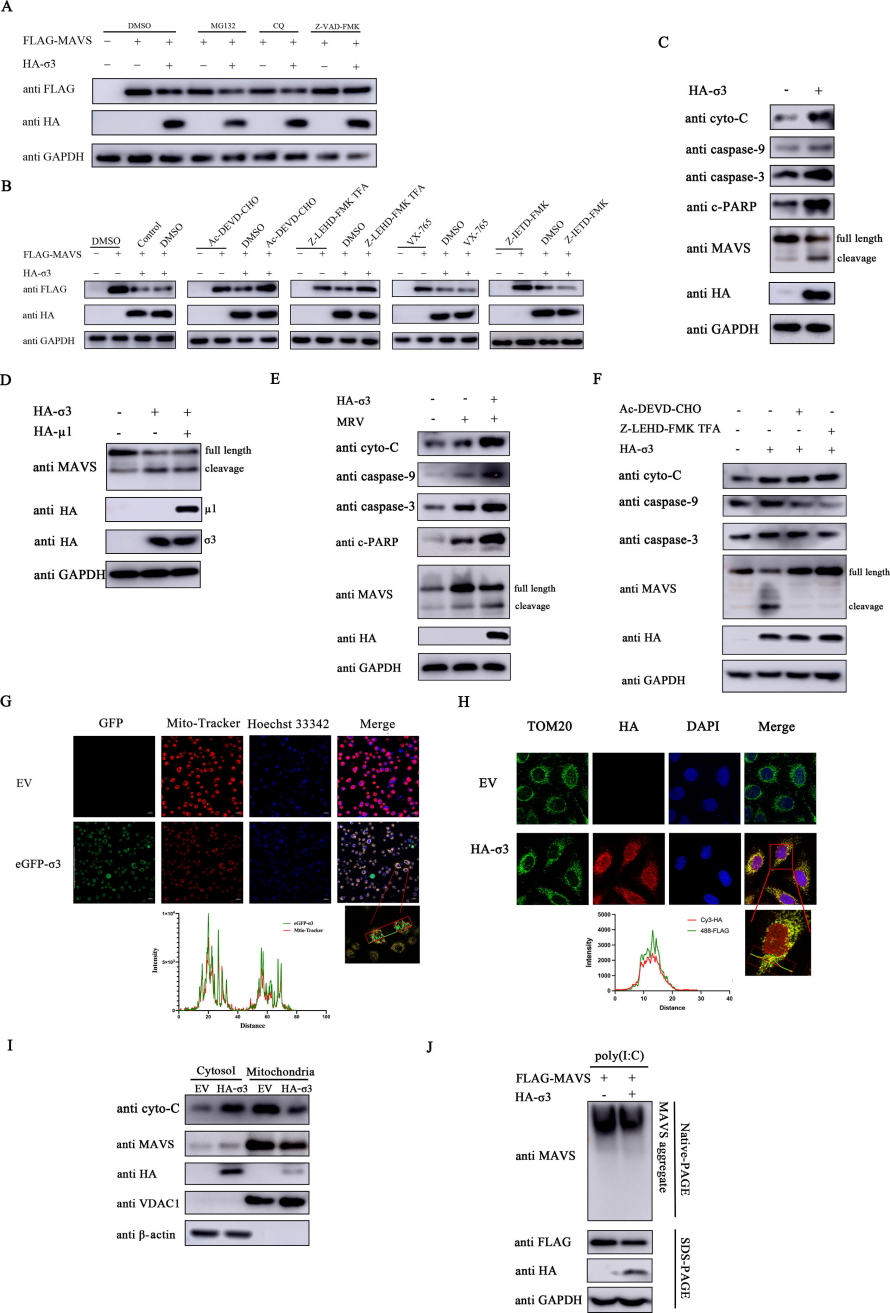
MRV σ3 protein degrades MAVS through the intrinsic apoptotic pathway. (**A**) A549 cells were transfected with 1.0 µg of EV or HA-σ3 plasmid and 1.0 µg of FLAG-MAVS plasmid for 24 h and then treated for 8 h with dimethyl sulfoxide (DMSO), MG132 (7.5 µM), CQ (50 µM), or Z-VAD-FMK (20 µM), respectively. Western blotting was used to detect the expression of HA-tagged σ3 protein and FLAG-tagged MAVS. GAPDH was used as a loading control. (**B**) A549 cells were transfected with 1.0 µg of EV or HA-σ3 plasmid and 1.0 µg of FLAG-MAVS plasmid for 24 h and then treated for 8 h with DMSO, Ac-DEVD-CHO (20 µM), Z-LEHD-FMK TFA (20 µM), VX-765 (20 µM), or Z-IETD-FMK (20 µM), respectively. Western blotting was used to detect the expression of HA-tagged σ3 protein and FLAG-tagged MAVS. GAPDH was used as a loading control. (**C**) A549 cells were transfected with 1.0 µg of EV or HA-σ3 plasmid for 24 h, respectively. Western blotting was used to detect the protein expression of cyto-C, caspase-9, caspase-3, cleaved PARP, MAVS, and HA-tagged σ3, respectively. GAPDH was used as a loading control. (**D**) A549 cells were transfected with either 1.0 µg of EV, HA-σ3, or HA-σ3 + HA-μ1 plasmid for 24 h, respectively. Western blotting was used to detect the protein expression of MAVS, HA-tagged μ1 and σ3 protein, respectively. GAPDH was used as a loading control. (**E**) A549 cells were transfected with 1.0 µg of EV or HA-σ3 plasmid for 24 h followed by MRV infection (MOI = 0.01) for 24 h. Western blotting was used to detect the protein expression of cyto-C, caspase-9, caspase-3, cleaved PARP, MAVS, and HA-tagged σ3, respectively. GAPDH was used as a loading control. (**F**) A549 cells were transfected with 1.0 µg of EV or HA-σ3 plasmid for 24 h and then treated for 8 h with DMSO, Ac-DEVD-CHO (20 µM), or Z-LEHD-FMK TFA (20 µM), respectively. Western blotting was used to detect the protein expression of cyto-C, caspase-9, caspase-3, MAVS, and HA-tagged σ3, respectively. GAPDH was used as a loading control. (**G**) A549 cells were transfected with 1.0 µg of EV or enhanced green fluorescent protein (eGFP)-σ3 plasmid. After 24 h, the cells were stained with Mito-Tracker and Hoechst 33342, followed by fluorescence analysis. Scale bar = 20 µm. (**H**) A549 cells were transfected with 1.0 µg of EV or HA-σ3 plasmid for 24 h before fixing cells and adding with corresponding antibodies for immunofluorescence imaging under a confocal microscope for HA (red), nucleus (blue), and TOM20 (green). Scale bar = 20 µm. (**I**) A549 cells were transfected with 1.0 µg of EV or HA-σ3 plasmid for 24 h. Then cells were collected and mitochondrial and cytosolic fractions were isolated using the Cell Mitochondria Isolation Kit. Western blotting was used to detect the expression of cyto-C, MAVS, and HA-tagged σ3 protein in the mitochondria and cytosol, respectively. VDAC1 was used as a mitochondrial loading control, whereas β-actin was used as a cytosolic loading control. (**J**) A549 cells were co-transfected with 1.0 µg of EV or HA-σ3 plasmid and 1.0 µg of FLAG-MAVS for 24 h, followed by transfection with 1.0 µg of poly(I:C) for another 12 h. The cells were treated with nondenaturing lysis buffer, part of the lysate was subjected to native-PAGE and the other part was subjected to SDS-PAGE. Western blotting was used to detect the oligomerization of MAVS, the expression of FLAG-tagged MAVS protein, and HA-tagged σ3 protein.

We speculate that σ3-mediated direct inhibition of MAVS occurs via a host degradative mechanism. To dissect this, we treated exogenous σ3-expressing cells with various inhibitors, including proteosomal inhibitor (MG132), autophagy inhibitor (chloroquine, CQ), and pan-caspase inhibitor (Z-VAD-FMK). Immunoblotting analysis revealed that σ3-mediated MAVS degradation was only blocked by Z-VAD-FMK (Fig. 6A), suggesting that apoptotic caspases are involved in this process. Specifically, the inhibition of exogenously expressed MAVS by σ3 was reversed in cells treated with caspase-3 inhibitor (Ac-DEVD-CHO) and caspase-9 inhibitor (Z-LEHD-FMK TFA) (Fig. 6B), indicating that this is occurring through the mitochondria-mediated intrinsic apoptosis pathway. Indeed, exogenously expressed σ3 elevated the expression of various key mediators in this pathway (cytochrome C, caspase-9, caspase-3, and cleaved poly ADP-ribose polymerase (c-PARP) (Fig. 6C). As apoptotic caspases, particular caspase-3, have been shown to cleave MAVS (27), we observed MAVS cleavage was occurring in parallel to the observed caspase-3 upregulation (Fig. 6C). Moreover, the degradative ability of σ3 is retained in cells co-expressed with MRV μ1 (Fig. 6D), suggesting that μ1 protein did not affect the degradation ability of σ3 on MAVS. Importantly, MRV infection also increased cytochrome C, caspase-9, caspase-3, and cleaved PARP levels, and this increase was additive in σ3-expressing cells (Fig. 6E). We further confirmed that Ac-DEVD-CHO and Z-LEHD-FMK TFA can also block σ3-mediated cleavage of endogenously expressed MAVS (Fig. 6F), highlighting the critical role of caspase-3 and caspase-9 in this process.

Considering these findings and that MAVS is associated with the mitochondria (28), we surmised that σ3-mediated degradation is occurring in the mitochondrial compartment. We confirmed that σ3 subcellularly localizes in the mitochondria with the aid of a mitochondrial probe (Mito-Tracker) and marker (TOM20) by immunofluorescence (Fig. 6G and H) and immunoblotting (Fig. 6I). The caspase-9 and caspase-3 axis is activated upon the release of cytochrome C from the mitochondria into the cytosol (29). Indeed, we observed decreased levels of cytochrome C in the mitochondria coinciding with an increase in cytosolic cytochrome C levels in σ3-expressed cells (Fig. 6I). Parallel to this, the decrease in levels of MAVS in the mitochondrial membrane and elevation in the cytosol was also observed. MAVS is known to undergo oligomerization during RLR signaling activation (30). We wanted to explore whether σ3 has a regulatory effect on this process. Native PAGE showed that σ3 significantly inhibited the oligomerization of MAVS (Fig. 6J). Overall, these above results demonstrate that σ3 co-localizes with MAVS in the mitochondria and promotes the mitochondria-mediated intrinsic apoptotic pathway to cleave and consequently degrade MAVS via caspase-3 and caspase-9.

### The tryptophan on position 133 of σ3 protein is required MAVS degradation

**Fig 7:**
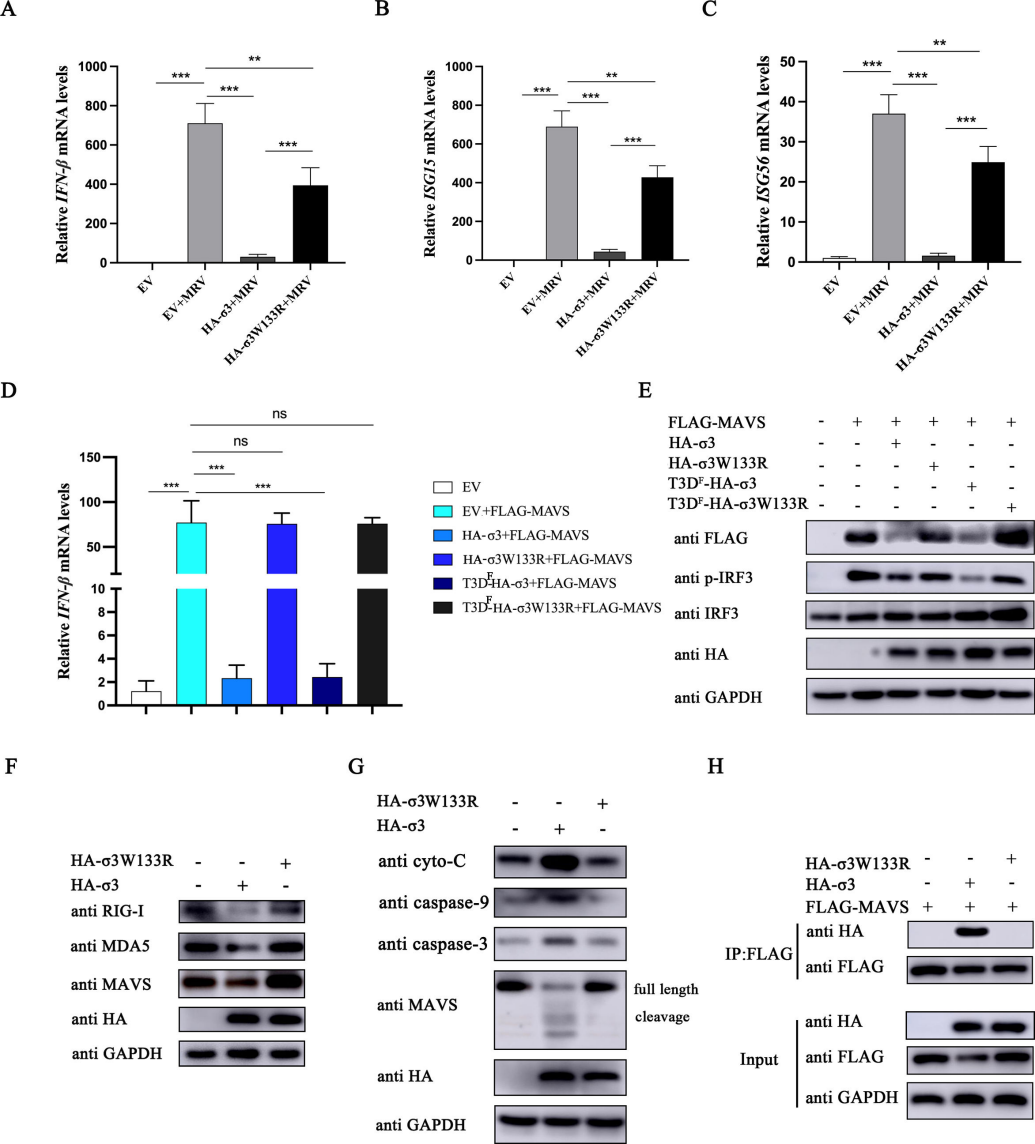
The tryptophan on position 133 of σ3 protein is a key residue for MAVS degradation. (**A–C**) A549 cells were transfected with 1.0 µg of EV, HA-σ3, or HA-σ3W133R plasmids for 24 h followed by MRV infection (MOI = 0.01) for 24 h. Total RNA was extracted and qPCR was used to detect the mRNA expression level of *IFN-*β, *ISG15*, and *ISG56*. (**D**) A549 cells were co-transfected with 1.0 µg of EV, HA-σ3, HA-σ3W133R, T3D^F^-HA-σ3, or T3D^F^-HA-σ3W133R along with FLAG-MAVS for 24 h, respectively. Total RNA was extracted and qPCR was used to detect the mRNA expression level of *IFN-*β. (**E**) A549 cells were co-transfected with 1.0 µg of EV, HA-σ3, HA-σ3W133R, T3D^F^-HA-σ3, or T3D^F^-HA-σ3W133R along with FLAG-MAVS for 24 h, respectively. Western blotting was used to detect the expression of FLAG-tagged MAVS, IRF3, p-IRF3, HA-tagged wild-type σ3 protein, and its mutant protein, respectively. GAPDH was used as a loading control. (**F**) A549 cells were transfected with 1.0 µg of EV, HA-σ3, or HA-σ3W133R plasmids for 24 h. Western blotting was used to detect the protein expression of endogenous RIG-I, MDA5, MAVS, and HA-tagged σ3, respectively. GAPDH was used as a loading control. (**G**) A549 cells were transfected with 1.0 µg of EV, HA-σ3, or HA-σ3W133R plasmids for 24 h. Western blotting was used to detect the protein expression of cyto-C, caspase-9, caspase-3, MAVS, HA-tagged wild-type σ3 protein, and its mutant protein, respectively. GAPDH was used as a loading control. (**H**) A549 cells were transfected with 1.0 µg of EV, HA-σ3, or HA-σ3W133R along with FLAG-MAVS for 24 h, respectively. The cells were collected and lysed, protein G was added to adsorb the proteins in the cell lysate, and then immunoprecipitated with FLAG antibody. Western blotting was used to detect FLAG-tagged MAVS, HA-tagged wild-type σ3 protein, and its mutant protein in the Input and IP complexes, respectively. The data were represented as the mean ± SD of three independent experiments. The measurements were performed in technical duplicate. Statistical significance was denoted as **P < 0.01 and ***P < 0.001.

A previous study demonstrated that the mutation of the 133rd amino acid of σ3 protein of MRV T3D^F^ strain from tryptophan (W) to arginine (R) diminished the inhibitory effect of σ3 on the NF-κB signaling pathway (13). In order to investigate whether this residue is essential for MRV σ3-mediated MAVS degradation, we overexpressed the HA-tagged σ3 mutant plasmid (HA-σ3W133R) and infected cells with MRV to see whether σ3-antagonizing I-IFN responses are impacted. The mutant σ3W133R protein significantly restored I-IFN responses (Fig. 7A through C) compared to the wild-type σ3 protein. To further confirm whether the tryptophan residue of σ3 protein derived from other MRV strains has inhibitory effects on MAVS-mediated IFN-β expression and the expression of key adaptor molecules in the MAVS-mediated RLR signaling pathway, we co-transfected wild-type σ3 plasmids or their mutant counterparts derived from MRV T3A and T3D^F^ strains with FLAG-tagged MAVS. Wild-type σ3 protein derived from both MRV strains significantly inhibited MAVS-mediated *IFN-*β expression, while their mutated counterparts did not (Fig. 7D). Moreover, the wild-type forms of σ3, but not its mutant versions, from both strains inhibited the expression of exogenous MAVS and phosphorylated IRF3 activated by MAVS (Fig. 7E). We also confirmed that mutant σ3W133R protein have lost the ability to inhibit the expression of endogenous RIG-I, MDA5, and MAVS (Fig. 7F). These results further confirm that MRV σ3, irrespective of which MRV strain it is derived from, has a universal ability to inhibit MAVS-mediated RLR signaling. Since σ3-mediated MAVS degradation is dependent on the cleavage of MAVS by the caspase-3 and caspase-9 (Fig. 6B and F), we wondered whether this cleavage process is dampened when σ3 is mutated. As speculated, the mutant σ3W133R protein has completely lost the ability to cleave MAVS and to upregulate cytochrome C, caspase-9, and caspase-3 expression (Fig. 7G). Moreover, mutant σ3 protein also failed to maintain its biochemical interaction with MAVS (Fig. 7H). Collectively, our findings demonstrate that tryptophan at position 133 of σ3 protein is a key residue that interacts with and degrades MAVS.

## DISCUSSION

It is widely recognized that RNA viruses infect cells by activating the RLR signaling pathways, thereby inducing the production of IFNs. The synergistic effect of the RLR signaling pathway-related adaptor molecules RIG-I, MDA5, MAVS, TBK1, and IRF3 form the first line of defense against RNA viruses (31). Viral proteins are known to target and inhibit RLR signaling pathway adaptor molecules, interfering with the production of IFNs, thus promoting virus replication. For example, EMCV VP2 protein (32) and coxsackievirus 2C protein (33) antagonize the IFN-β responses by targeting RLR signaling components, while the nucleocapsid protein of peste des petits ruminants virus inhibits IFN production by directly interacting with IRF3 (34). For MRV, its uNS protein has been shown to dampen IFN production by sequestering IRF3 into viral factories (35), whereas μ2 protein was shown to inhibit ISG expression by sequestering IRF9 in the nucleus (36). However, little is known about the antagonism of innate immune responses by other viral proteins of reoviruses. In this study, we demonstrate that MRV σ3 protein can block RLR signaling transduction by directly degrading MAVS (Fig. 4).

The distribution of the σ3 protein of different strains of MRV in cells was different, which was likely due to the different affinity between the σ3 protein of different strains and the μ1/μ1C protein. This result is consistent with the study by Schmechel et al. (37). We found that the distribution of heterologous expression of σ3 protein was also inconsistent with that of virus infection. The heterologous expression of T3A strain σ3 protein was mainly distributed in the nucleus, with some diffuse distribution in the cytosol (Fig. 4I). However, after infection with the MRV T3A strain, the σ3 protein was mainly distributed in the perinuclear region and cytosol (Fig. 4J). This may be due to the interaction between σ3 and the μ1/μ1C, which forms a complex that blocks the migration of σ3 to the nucleus (24). In addition, we found that both the heterologous expressions of the σ3 protein and the σ3 protein during viral infection were both co-localized with MAVS (Fig. 4I and J). Though σ3 protein would complex with MRV μ1 to form a heterohexamer during MRV infection (8, 9), we found that σ3 protein retains the capacity to interact with and degrade MAVS in the presence of μ1 (Fig. 4H and 6D). Since MAVS is specifically localized to the mitochondrial outer membrane, we confirm that MRV σ3 protein also co-localized with the mitochondria (Fig. 6G and H). These results all demonstrate the reliability of σ3 targeting MAVS and co-locating with MAVS in the mitochondria.

MAVS is known to have a regulatory effect on the expression of RIG-I and MDA5 (25). In this study, we found that MAVS has a positive feedback effect on the expression of RIG and MDA5, and that the degradation of RIG-I and MDA5 by σ3 protein occurs independently of its ability to inhibit MAVS (Fig. 5), suggesting that MRV σ3 protein can inhibit the activation of RLR/MAVS signaling pathway through two different mechanisms enabling MRV to evade the host’s innate immune response and thus facilitate its own replication. The interaction between viruses and mitochondria plays an important role in the pathogenic mechanism of viruses (38–40). Here, we show that MRV infection can induce apoptosis, and that MRV σ3 promotes the release of cytochrome C from the mitochondria into the cytosol, further activating downstream apoptotic mediators (Fig. 6C, E, and I). Caspase-3 is known to cleave MAVS (27), and demonstrated that MAVS degradation by σ3 protein is dependent on the bioactivity of caspase-3 and caspase-9 (Fig. 6F). Our findings also suggest that σ3 protein can also localize into the mitochondria, and whether this influences the mitochondrial outer membrane potential to activate the mitochondrial apoptotic pathway remains to be studied. Although many viruses promote viral replication by inhibiting apoptosis, apoptosis can also limit the viral replication by inducing cellular death of infected cells (41, 42). When viruses infect cells, they initiate the apoptotic pathway which triggers cell death, and hence further replication and spread of the virus are limited. However, MRV σ3 appears to be limiting host antiviral innate immune response by hijacking the apoptotic pathway, thereby facilitating its own replication and transmission. In general, the relationship between apoptosis and viral replication is complex, which is regulated by the temporal and spatial network, and the effect of apoptosis on viral replication at a specific time is the result of the interaction and mutual game between viruses and host cells.

The expression of a single viral protein of the MRV T3D^F^ strain, σ3, was sufficient to inhibit the expression of NF-κB target gene and subsequent IFN activation (12, 13), while σ3 protein of the reovirus strain T3D^L^ (a T3D isolate from Patrick Lee’s laboratory) failed to block the expression of the TNF-α-induced NF-κB reporter system. Importantly, the polymorphic differences in the 133rd amino acid of σ3 protein affect its ability to inhibit NF-κB (13). Both T3A and T3D^F^ σ3 protein include a tryptophan at position 133, whereas the T3D^L^ σ3 protein has the arginine at this position. In this study, the T3A strain was used and it was found that the 133rd tryptophan residue σ3 is a key residue for MAVS inhibition, but may not be the sole residue for RLR signaling inhibition (Fig. 7). When the 133rd tryptophan residue of σ3 protein was mutated to arginine, its inhibition of MAVS was lost, but the inhibitory effect on the protein expression of RIG-I was not fully restored (Fig. 7F). Hence, further studies investigating the key domains and residues of σ3 protein inhibition of RIG-I are still needed. Although the σ3 protein has a dsRNA-binding domain (5), its 133rd amino acid does not fall within this domain. Roebke et al. found that the ability of σ3 protein to bind dsRNA does not affect its inhibitory effect on the IFN production (12). Our results are consistent with this, demonstrating that the σ3 protein inhibits the RLR signaling pathway through a mechanism independent of dsRNA binding.

**Fig 8:**
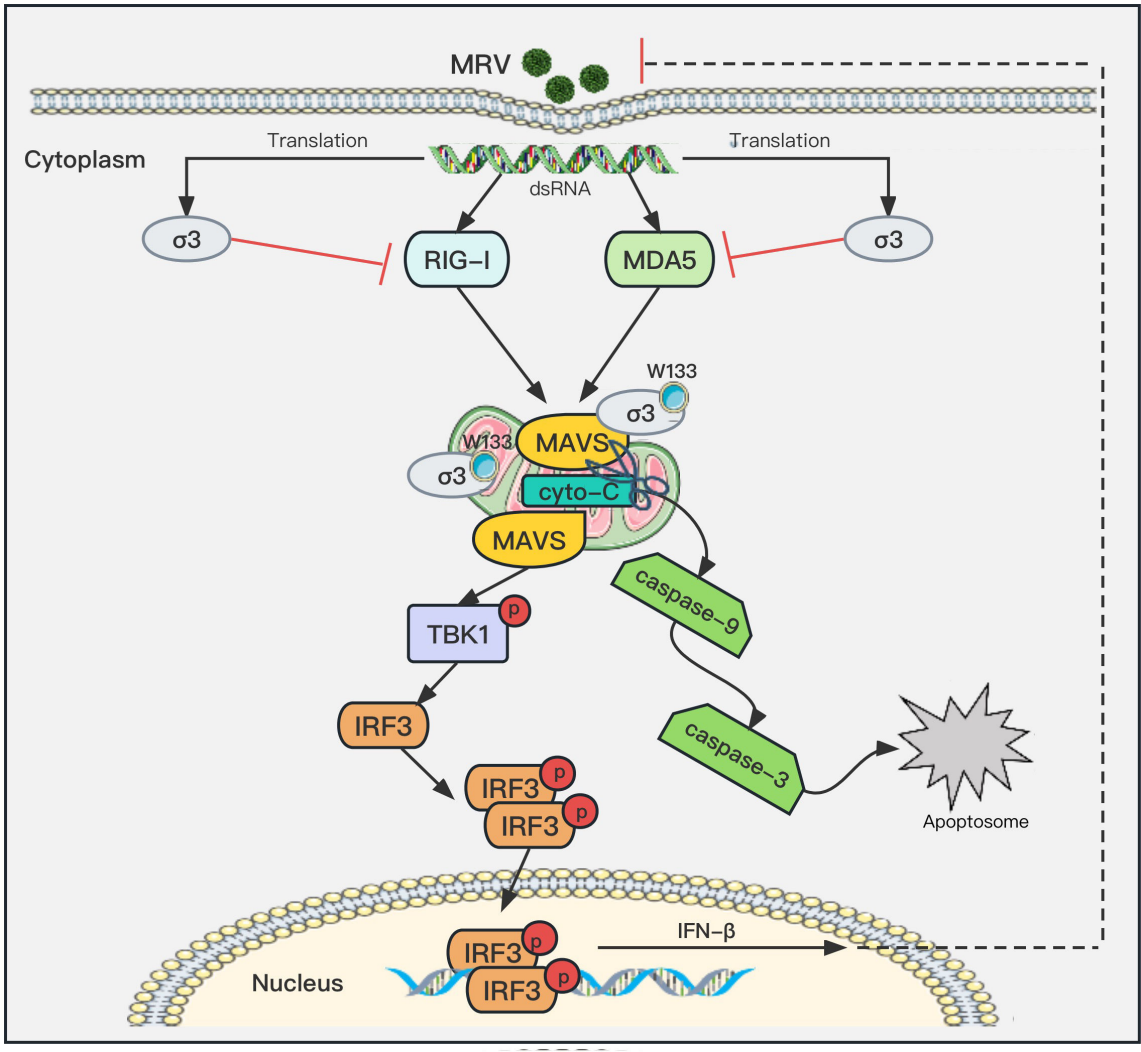
Schematic model of the MRV σ3 protein promotes viral proliferation by negatively regulate the RLR signaling pathway. MRV σ3 protein co-localizes with MAVS in mitochondria and interacts with the C-terminal domain of MAVS to cleave and subsequently degrade MAVS via the caspase-9- and caspase-3-dependent apoptotic pathway, thereby inhibiting the transduction of the RLR signaling pathway and ultimately promoting MRV replication *in vitro*. In addition, σ3 protein inhibits RIG-I and MDA5 expression is independent of its inhibitory effect on MAVS. The tryptophan on position 133 of σ3 protein plays a key role in the interaction and degradation of MAVS.

In summary, our results have revealed a mechanism by which MRV σ3 protein targets the degradation of MAVS through the caspase-9- and caspase-3-dependent mitochondrial apoptosis pathway, thereby inhibiting the RLR/MAVS signaling pathway (Fig. 8). This study provides a better understanding of the mechanisms by which MRV infection evades the host innate immune response to facilitate viral infection and transmission.

## MATERIALS AND METHODS

### Cells and viruses

HEK293 cells, BHK-21 cells, and A549 cells were obtained from the American Type Culture Collection and maintained at a 37°C incubator containing 5% CO_2_. HEK293 cells and BHK-21 cells were cultured in Dulbecco's modified Eagle medium (DMEM), supplemented with 10% new bovine serum (NBS) (Lanzhou Minhai, Lanzhou, China). A549 cells were cultured in F12 medium containing 15% NBS. HEK293T-Vector cells and HEK293T-KO-MAVS cells were cultured at 37°C under 5% CO_2_ incubator in DMEM supplemented with 10% fetal bovine serum (Lanzhou Minhai, Lanzhou, China). MRV strain Abney (T3A), EMCV strain PV21 (GenBank No: X74312), and VSV-GFP were provided by Biomedical Research Center of Northwest Minzu University.

### Plasmids, reagents and antibodies

Expression plasmids FLAG-RIG-I, FLAG-MDA5, FLAG-MAVS, FLAG-CTD, FLAG-PRO, FLAG-CARD, FLAG-TBK1, and FLAG-IRF3(5D) were all constructed in our laboratory. T3A-HA-σ3 (HA-σ3 for short), T3A-eGFP-σ3 (eGFP-σ3 for short), T3A-HA-σ3W133R (HA-σ3W133R for short), T3D^F^-HA-σ3, and T3D^F^-HA-σ3W133R plasmids were synthesized by Genscript (Nanjing, Jiangsu, China). Lipofectamine 2000 was purchased from Invitrogen (Waltham, MA, USA). Anti-FLAG, anti-HA, anti-Myc, anti-IRF3, anti-RIG-I, anti-MDA5, anti-MAVS, anti-caspase-9, anti-caspase-3, anti-cyto-C, anti-GAPDH, anti-VDAC1, anti-TOM20, and anti-β-actin antibodies were purchased from Proteintech (Wuhan, Hubei, China). Anti-TBK1, anti-p-TBK1, and anti-p-IRF3 antibodies were purchased from Cell Signaling Technology (Boston, MA, USA). Anti-c-PARP antibody, MG132, Z-VAD-FMK, Ac-DEVD-CHO, NP-40 lysis buffer, radio-immunoprecipitation assay lysis buffer (RIPA lysis buffer), Mito-Tracker Red CMXRos, and Cell Mitochondria Isolation Kit were purchased from Beyotime (Shanghai, China). Reovirus σ3 mouse antibody (4F2) was purchased from Developmental Studies Hybridoma Bank (Iowa, IA, USA). Polyinosinic-polycytidylic acid and CQ were purchased from InvivoGen (San Diego, CA, USA). Hoechst 33342 was purchased from Solarbio (Beijing, China). Z-IETD-FMK, Z-LEHD-FMK TFA, and VX-765 were purchased from MedChemExpress (Monmouth Junction, NJ, USA).

### Cell transfection and Western blotting

HEK293, A549, HEK293T-Vector, or HEK293T-KO-MAVS cells in six-well plates or 10 cm dishes were transfected with indicated expression plasmids using Lipofectamine 2000 (Invitrogen, Waltham, MA, USA) for 24 h, respectively. HEK293 or A549 cells in six-well plates or 10 cm dishes were transfected with poly(I:C) (InvivoGen, San Diego, CA, USA) using Lipofectamine 2000 for 12 h, respectively. Cells were collected and lysed with RIPA buffer containing protease and phosphatase inhibitors (Beyotime, Shanghai, China). Equal amounts of cell extracts were denatured, run on 8% or 10% SDS-PAGE gels, and transferred to polyvinylidene fluoride (PVDF) membranes (Millipore, MA, USA). The membranes were blocked with 2.5% skimmed milk in Tris-buffered saline and Tween 20 (TBST) (Solarbio, Beijing, China) for 2 h at room temperature, then incubated with specific primary antibodies (1:1,000) for 6–8 h at 4°C and secondary antibodies (1:10,000) for 1 h at room temperature, respectively. Finally, proteins on membranes were detected using Western lightning Plus-ECL kit (PerkinElmer, Waltham, MA, USA).

### Virus infectivity assays

For *in vitro* virus infection, treated or untreated HEK293 cells or A549 cells were washed with phosphate buffered saline (PBS) three times and infected with MRV strain T3A (MOI = 1, 0.1 or 0.01), EMCV strain PV21 (MOI = 0.001), or VSV-GFP (MOI = 0.001) for 2 h, respectively. These cells were then continued to be cultured in maintenance medium for a certain period of time. The viral suspension was collected after freeze-thawing for three times, and the titers of different viruses were measured in BHK-21 cells by TCID_50_ method (Reed-Muench method).

### RNA extraction and quantitative PCR (qPCR)

Total intracellular RNA was extracted with TRIZOL reagent (Takara, Beijing, China). The extracted RNA was synthesized into cDNA using the Evo M-MLV Reverse Transcription Premix Kit (Accurate Biology, Hunan, China). The mRNA expression levels of *IFN-*β, *ISG15,* and *ISG56* were detected using TransStart Top Green qPCR SuperMix (+Dye II) (Transgen, Beijing, China). The relative abundances of target genes were normalized to that of GAPDH by the 2^−∆∆Ct^ method. The qPCR primers are shown in Table 1.

**TABLE 1:** The sequences of qPCR primers used in this study

Primer	Sequence (5´ to 3´)
Homo-*IFN-*β-qF	GTCAGAGTGGAAATCCTAAG
Homo-*IFN-*β-qR	ACAGCATCTGCTGGTTGAAG
Homo-*ISG15*-qF	AGGACAGGGTCCCCCTTGCC
Homo-*ISG15*-qR	CCTCCAGCCCGCTCACTTGC
Homo-*ISG56*-qF	ACGGCTGCCTAATTTACAGC
Homo-*ISG56*-qR	AGTGGCTGATATCTGGGTGC
Homo-*GAPDH*-qF	GTCTCCTCTGACTTCAACAGCG
Homo-*GAPDH*-qR	ACCACCCTGTTGCTGTAGCCAA

### siRNA interference assay

When the density of A549 cells reached 30%–40%, siRNA oligos was transfected into the cells with Lipofectamine 2000, and siNC was used as the negative control. siRNA was synthesized by RiboBio (Guangzhou, China). The siRNA sequences are shown in Table 2.

**TABLE 2:** The siRNA sequences used in this study

Target gene	Sequence (5´ to 3´)
siσ3-001	CGCGTTCAATGGTGTGAAA
siσ3-002	CTGGAGCATGATCCATTGA
siσ3-003	CGGCTAAGTTGAAGACAGT
siRNA-MAVS	CCACCTTGATGCCTGTGAA, CAGAGGAGAATGAGTATAA
siNC	GTTCTCCGAACGTGTCACGT

### Immunoprecipitation

The transfected cells were collected and lysed using NP-40 buffer containing protease inhibitors (Beyotime, Shanghai, China). A portion of the cell lysate was used for Input following the protein immunoblotting method described above. Another portion was used as IP to adsorb the proteins in the cell lysate using protein G (Beyotime, Shanghai, China), followed by immunoprecipitation using the indicated antibodies. Immunoprecipitated samples were gathered and utilized for Western blotting.

### ELISA assay

Human IFN-β ELISA Kit (Shanghai Enzyme-Linked Biotechnology, Shanghai, China) was used to detect the expression of IFN-β in the cell supernatant according to the manufacturer’s instructions.

### Inhibitor treatment assay

A549 cells in six-well plates were transfected with pCMV-HA (EV), HA-σ3, and other recombinant plasmids using Lipofectamine 2000. At 24 h post-transfection, the cells were treated with pan-caspase inhibitor Z-VAD-FMK (Beyotime, Shanghai, China), proteasome inhibitor MG132 (Beyotime, Shanghai, China), endosomal acidification and autophagy inhibitor CQ (InvivoGen, San Diego, CA, USA), caspase-3 inhibitor Ac-DEVD-CHO (Beyotime, Shanghai, China), caspase-8 inhibitor Z-IETD-FMK (MedChemExpress, Monmouth Junction, NJ, USA), caspase-9 inhibitor Z-LEHD-FMK TFA (MedChemExpress, Monmouth Junction, NJ, USA), caspase-1 inhibitor VX-765 (MedChemExpress, Monmouth Junction, NJ, USA), or DMSO for 8 h, respectively. Then, the cells were collected and lysed with RIPA lysis buffer supplemented with protease and phosphatase inhibitors. Equal amounts of cell extracts were denatured and analyzed using 10% SDS-PAGE gels, and then transferred to methanol-activated PVDF membranes. Subsequently, these membranes were blocked with 2% bovine serum albumin or 2.5% skim milk before specific primary and secondary antibodies were added, respectively. Finally, proteins on membranes were detected using ECL Substrate (Hercules, CA, USA).

### Indirect immunofluorescence

Cells cultured on Nunc glass-bottom petri dishes (ThermoFisher Scientific, Waltham, MA, USA) were transfected with various plasmids or infected with VSV. Cells were fixed with 4% paraformaldehyde for 10 min and blocked with 1% PBST bovine serum albumin for 6–8 h at 4°C. Cells were then incubated with a 1:250 dilution of appropriate primary antibody overnight at 4°C, followed by incubation with a 1:500 dilution of Cy3-conjugated goat anti-mouse IgG and/or CoraLite488-conjugated goat anti-rabbit IgG (Jackson ImmunoResearch Laboratories, West Grove, PA, USA) for 1 h at room temperature in the dark. Cells were then stained with 4´,6-diamino-2-phenylindole for 10 min at room temperature to reveal the nuclei. Fluorescence was observed by ZEISS LSM900 laser confocal microscopy.

### Mitochondrial isolation and mitochondrial co-localization

A549 cells were transfected with the expression plasmids, and after 24 h of cell culture, the mitochondria were separated using the Cell Mitochondria Isolation Kit (Beyotime, Shanghai, China), and then were subjected to Western blotting assay. Cells cultured on Nunc glass-bottomed Petri dishes (ThermoFisher Scientific, Waltham, MA, USA) were transfected with the expression plasmid and stained with Mito-Tracker Red CMXRos (Beyotime, Shanghai, China) for 30 min after 24 h. Hoechst 33342 (Solarbio, Beijing, China) was then added to stain the nuclei for 10 min to reveal the nuclei. The fluorescence was observed by ZEISS LSM900 laser confocal microscope.

### Statistical analysis

Data are expressed as the mean ± SD. Statistical significance was determined by using Student’s two-tailed non-parametric *t*-test or analysis of variance with GraphPad Prism software (version 6.0, USA). Differences between groups were considered significant when the *P* value was <0.05 (*), <0.01 (**), and <0.001 (***).

## Data Availability

The data that support the findings of this study are available from the corresponding authors upon request.
